# Genome-Wide Identification and Characterization of *NODULE-INCEPTION-Like Protein (NLP)* Family Genes in *Brassica napus*

**DOI:** 10.3390/ijms19082270

**Published:** 2018-08-02

**Authors:** Miao Liu, Wei Chang, Yonghai Fan, Wei Sun, Cunmin Qu, Kai Zhang, Liezhao Liu, Xingfu Xu, Zhanglin Tang, Jiana Li, Kun Lu

**Affiliations:** 1 College of Agronomy and Biotechnology, Southwest University, Beibei, Chongqing 400715, China; monky1117@email.swu.edu.cn (M.L.); fyh1212@email.swu.edu.cn (Y.F.); reginasw@163.com (W.S.); drqucunmin@swu.edu.cn (C.Q.); kaizhang2013@gmail.com (K.Z.); liezhao2003@126.com (L.L.); xinfuxu@126.com (X.X.); tangzhlin@swu.edu.cn (Z.T.); ljn1950@swu.edu.cn (J.L.); 2 Academy of Agricultural Sciences, Southwest University, Beibei, Chongqing 400715, China; cw12345678@email.swu.edu.cn; 3 Shennong Class, Southwest University, Beibei, Chongqing 400715, China

**Keywords:** NODULE-INCEPTION-like protein, *Brassica napus*, evolution, nitrogen deficiency

## Abstract

NODULE-INCEPTION-like proteins (NLPs) are conserved, plant-specific transcription factors that play crucial roles in responses to nitrogen deficiency. However, the evolutionary relationships and characteristics of *NLP* family genes in *Brassica napus* are unclear. In this study, we identified 31 *NLP* genes in *B. napus*, including 16 genes located in the A subgenome and 15 in the C subgenome. Subcellular localization predictions indicated that most BnaNLP proteins are localized to the nucleus. Phylogenetic analysis suggested that the *NLP* gene family could be divided into three groups and that at least three ancient copies of *NLP* genes existed in the ancestor of both monocots and dicots prior to their divergence. The ancestor of group III *NLP* genes may have experienced duplication more than once in the Brassicaceae species. Three-dimensional structural analysis suggested that 14 amino acids in BnaNLP7-1 protein are involved in DNA binding, whereas no binding sites were identified in the two RWP-RK and PB1 domains conserved in BnaNLP proteins. Expression profile analysis indicated that *BnaNLP* genes are expressed in most organs but tend to be highly expressed in a single organ. For example, *BnaNLP6* subfamily members are primarily expressed in roots, while the four *BnaNLP7* subfamily members are highly expressed in leaves. *BnaNLP* genes also showed different expression patterns in response to nitrogen-deficient conditions. Under nitrogen deficiency, all members of the *BnaNLP1/4/5/9* subfamilies were upregulated, all *BnaNLP2/6* subfamily members were downregulated, and *BnaNLP7/8* subfamily members showed various expression patterns in different organs. These results provide a comprehensive evolutionary history of *NLP* genes in *B. napus*, and insight into the biological functions of *BnaNLP* genes in response to nitrogen deficiency.

## 1. Introduction

Nitrogen is a primary nutrient that is critical for the survival of all living organisms. The absorption and utilization of nitrogen directly affects plant growth and development, as well as crop yields. During their long evolutionary history, plants have established complex and delicate regulatory mechanisms to adapt to changes in nitrogen conditions in the external environment. The main forms of nitrogen in soil include nitrate nitrogen, ammonium nitrogen, amino acids, proteins, and other nitrogenous substances. Nitrate nitrogen, the major form of nitrogen in soil, plays important physiological and nutritional roles in plant growth and development [1]. Nitrate nitrogen uptake and translocation in plant roots are mainly accomplished by nitrate nitrogen transporters (NRT) [2]. The first transporter identified to sense nitrate was the NRT1.1 in *Arabidopsis thaliana*, and the CIPK23 (a protein kinase) and CIPK8 can phosphorylate NRT1.1 to regulate the high- or low-affinity nitrate response [3,4]. Meanwhile, several proteins are involved in nitrogen assimilation in plants exposed to changing nitrogen concentrations, such as ammonium transporters (AMTs) and nitrate reductases (NRs) [5,6,7]. Genes and transcription factors (TFs) involved in nitrate-signaling pathways play key roles in nitrate absorption and assimilation [8]. *NIN* (NODULE INCEPTION) genes were first discovered as being defective in bacterial recognition, infection thread formation, and nodule primordia initiation in the lotus (*Lotus japonicus*), and the formation of rhizobia in leguminous species was later confirmed to be dependent on the presence of *NIN* genes [9,10]. *NIN* genes encode nuclear-targeted DNA binding proteins with bZIP domains and the most obvious feature of the NIN proteins is a strongly conserved 60-amino acid (aa)-long sequence, known as the RWP-RK (also known as the RWPxRK motif) sequence. Moreover, a few genes that possessed high degree homology with *NIN* were identified in legumes, named *NLP* (NIN-like proteins) [10,11,12]. Subsequent studies found that *NLP* genes widely exist among non-nitrogen fixating plants, such as rice (*Oryza sativa*) and *A. thaliana* [10], but the NIN proteins seem to be unique in legumes.

The *NLP* family is a plant-specific TF family, their proteins presented a homology to NIN not only in the RWP-RK domain but also in N-terminal regions [10,13]. NLP proteins contain two major conserved domains, the RWP-RK and PB1 (Phox and Bem1) domains. The RWP-RK domain functions in the DNA binding domain whose activity is unrelated to nitrate signaling, whereas the N-terminal regions of NLPs function as a transcriptional activation domain that mediates this signaling, and the PB1 domain is involved in protein–protein interactions [14,15]. A highly conserved GAF domain (a ubiquitous signaling motif and a new class of cyclic GMP receptor, which ensures the normal functioning of photosensitizing light reversals and optical signal transduction [3]) was also identified in the nitrogen termini of some NLP proteins, and may be related to signal transduction or dimerization [3,16,17]. *A. thaliana* contains nine *NLP* genes [8], whereas lotus contains four *NLPs* with a single *NIN* [13,18,19]. A number of *NLPs* have also been identified in maize (*Zea mays*) (nine), sorghum (*Sorghum bicolor*) (five), and rice (six) [10,14,20]. Functional studies have shown that *AtNLPs* play key roles in orchestrating primary nitrogen responses by binding to the nitrogen responsive *cis*-elements (NREs) in the promoters of target genes [9,21,22]. *AtNLP7* functions in nitrate-and nitrogen starvation responses by binding to key nitrogen pathway genes, including *ANR1*, *NRT1.1*, *NRT2*, and *LBD37/38*, thus moderating nitrogen assimilation and metabolism by transcriptionally activating or suppressing downstream genes [23,24]. *AtNLP8* regulates nitrate-promoted seed germination and directly binds to the promoter of an abscisic acid (ABA) catabolic enzyme gene to reduce ABA levels in a nitrate-dependent manner [19]. AtNLP6 and AtNLP7 proteins interact with the key transcriptional regulator TEOSINTE BRANCHED1/CYCLOIDEA/PROLIFERATING CELL FACTOR1-20 under nitrogen starvation and continuous nitrate treatment; these interacting regulators play a central role in controlling the expression of nitrate-responsive genes [25]. Nitrate triggers nitrate-CPK (Ca^2+^-sensor protein kinases)-NLP signaling and nitrate-coupled CPK signaling to phosphorylate NLPs, which play a crucial role in nutrient-growth networks [26].

*Brassica napus* is one of the most important oilseed crops in non-nitrogen fixating plants. This species was formed by the hybridization of two progenitor species, *Brassica rapa* and *Brassica oleracea* [27,28]. *NLP* genes play important roles in nitrogen uptake and utilization, which are critical for plant growth and development [10,14,20,29]. However, few studies have focused on identifying *NLP* genes in *B. napus*. In this study, we identified 31 *BnaNLP* genes using nine AtNLP proteins from *A. thaliana* as query sequences. We investigated the evolutionary relationships among NLP members from nine plant species and evaluated the predicted three-dimensional structures of a representative NLP protein (BnaNLP7-1) in *B. napus*. We also examined the spatio-temporal expression patterns of *BnaNLP* genes, as well as their expression under nitrogen deficient conditions, to reveal the relationship between *BnaNLPs* and nitrogen deficiency responses. Our study provides insight into the evolution of NLP proteins in plants and lays the foundation for elucidating the roles of *NLPs* in regulating nitrogen responses in *B. napus*.

## 2. Results

### 2.1. Identification of NLP Genes in B. napus

Using the nine AtNLP protein sequences in *A. thaliana* as queries, 31, 17, and 8 *NLP* genes were identified from *B. napus*, *B. rapa*, and *B. oleracea*, respectively. All *Brassica* NLP proteins share two conserved domains: The RWP-RK and PB1 domains. The nomenclature used for *Brassica NLP* genes was based on the corresponding *AtNLP* orthologs (Table 1 and Table S2). The number of amino acid residues in the BnaNLP proteins ranged from 681 (BnaNLP3-1) to 978 (BnaNLP7-3), with an average of 845. The relative molecular weights (MWs) ranged from 76.37 (BnaNLP3-1) to 108.05 kDa (BnaNLP7-3). The isoelectric points (pIs) were predicted to range from 4.98 (BnaNLP1-2) to 7.31 (BnaNLP3-1), and only two members had pI values > 7 (BnaNLP2-4 and BnaNLP3-1). No transmembrane helix or signal peptide was found in any BnaNLP protein (Table 1). Most BnaNLP proteins were predicted to localize in the nucleus, while BnaNLP2-4 was predicted to localize on the chloroplast. In addition, BnaNLP2-2 was predicted to be localized to the chloroplast and nucleus (Table 1), which is consistent with the general nuclear localization of TFs.

### 2.2. Phylogenetic Analysis of BnaNLP Proteins

**Figure 1:**
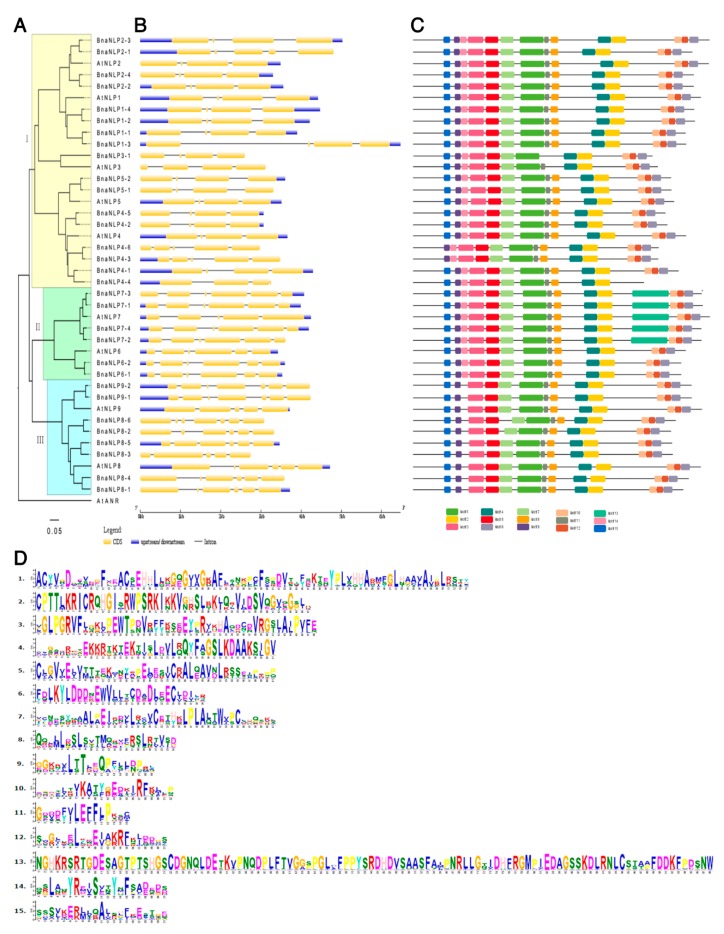
Phylogenetic relationships, gene structures, and conserved motifs of NLP proteins in *A. thaliana* and *B. napus*. The amino acid sequences of AtNLPs and BnaNLPs were aligned using ClustalW2. (**A**) The phylogenetic tree was constructed using MEGA 7.0 with the neighbor-joining method (1000 bootstrap replicates) and displayed using FigTree v1.4.0. The 40 NLP proteins in *A. thaliana* and *B. rapa* are clustered into three distinct groups. The scale bar represents 0.05 kb. At: *A. thaliana*; Bna: *B. napus*; (**B**) gene structures generated using the Gene Structure Display Server. Exons (CDS) and introns are shown with yellow wedges and black lines, respectively. Numbers 0, 1, and 2 represent introns in phases 0, 1, and 2, respectively; (**C**) conserved motifs of BnaNLP proteins identified by MEME. The motifs are indicated by colored boxes and their numbers are shown in the scale below the diagram; (**D**) sequences of the 15 conserved motifs in BnaNLPs identified in this study.

To clarify the evolutionary relationships between NLP proteins from *A. thaliana* and *B. napus*, we aligned the sequences of 40 NLP proteins using AtANR1 as an outgroup. Based on previous studies [8,10], NLP proteins were divided into three groups (Figure 1A), including the NLP1/2/3/4/5 clade (group I), the NLP6/7 clade (group II), and the NLP8/9 clade (group III). Group I contained 17 BnaNLPs, while groups II and III contained six and eight BnaNLPs, respectively. Both the BnaNLP4 and BnaNLP8 subfamilies contained six members, with more members compared to the other BnaNLP subfamilies.

**Figure 2:**
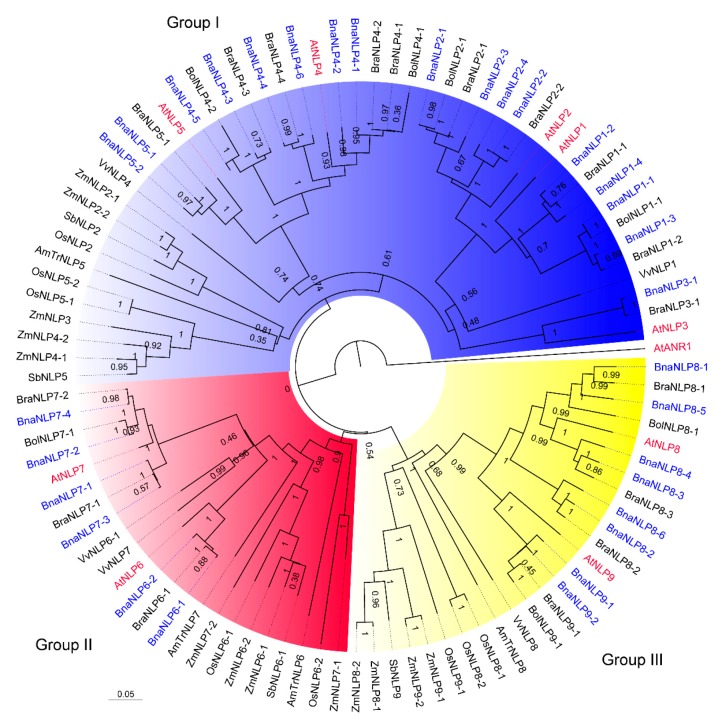
Phylogenetic relationships between BnaNLPs and other plant NLP proteins. The phylogenetic tree was constructed using MEGA 7.0 with the neighbor-joining method (1000 bootstrap replicates) and displayed using FigTree v1.4.0. NLP proteins in the phylogenetic tree are clustered into three distinct groups. At: *A. thaliana* (marked in red), Bra: *B. rapa*, Bol: *B. oleracea*, Bna: *B. napus* (marked in blue), AmTr: *A. trichopoda*, Os: *O. sativa*, Vv: *V. vinifera*, Zm: *Z. mays*, Sb: *S. bicolor*. Groups I, II and III are indicated by a blue, red, and yellow background, respectively.

To further investigate the evolutionary relationships of NLP proteins in plants, we constructed a larger neighbor-joining (NJ) tree based on the 99 NLP protein sequences from *A. thaliana*, *B. napus*, *B. rapa*, *B. oleracea*, *Amborella trichopoda*, rice, grape (*Vitis vinifera*), maize, and sorghum (Figure 2). The 99 plant NLP proteins were also classified into three groups. Group I was the largest clade, containing 49 NLP members, including 36 NLPs in Brassicaceae. Group II was the smallest clade, containing 23 NLP members, including 12 NLPs in Brassicaceae. Group III contained 26 NLP members, including 16 NLPs in Brassicaceae. BnaNLP members first clustered with NLPs from two progenitors of *B. napus*, followed by AtNLPs, forming a small Brassicaceae clade, and subsequently further clustered with NLP members in *V. vinifera* to form a dicot clade. Finally, a large clade could be observed by joining NLP proteins in dicot and monocot plants (Figure 2). In group I, the dicot clade was formed by two subclades, the NLP1/2/3 and NLP4/5 subclades. In the NLP1/2/3 subclade, NLP1/2 members in Brassicaceae first formed a small subclade clustered with VvNLP1 and then clustered with NLP3 members specifically in Brassicaceae. Plant NLP members were divided into three groups, with at least one NLP member present in *A. trichopoda*, suggesting that at least three ancient NLP copies were present in the ancestor before the monocot and dicot divergence.

### 2.3. Chromosome Location and Ka/Ks Ratio Calculation

**Figure 3:**
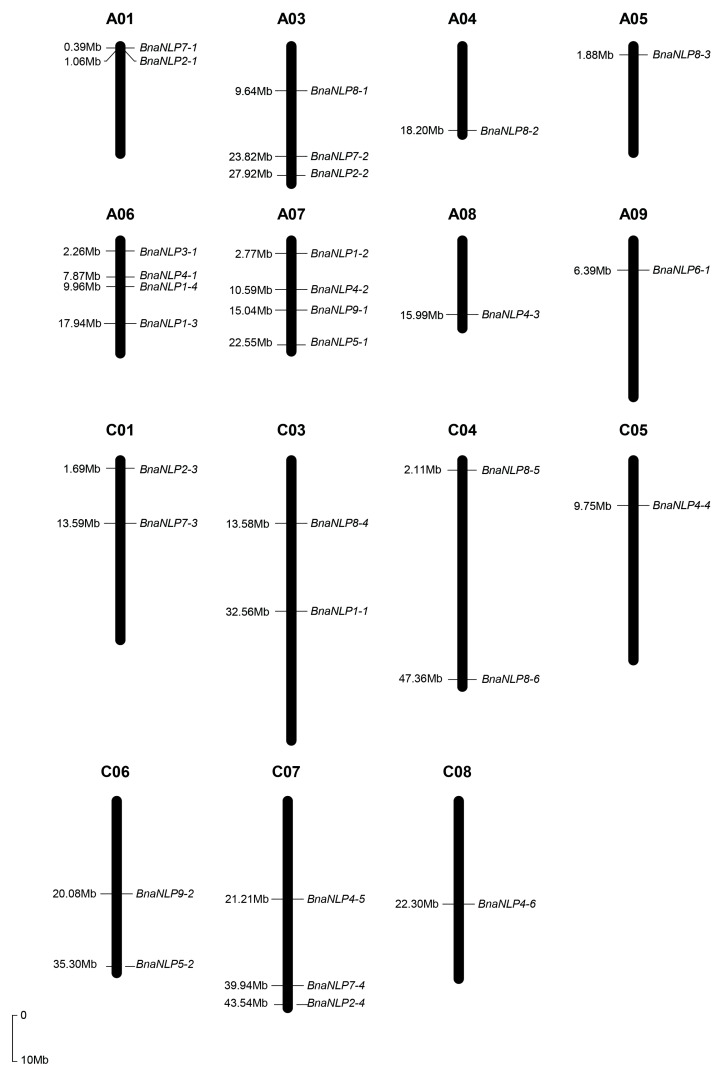
Chromosomal distribution of *BnaNLP* genes in the *B. napus* genome. Chromosomal information for *BnaNLP* genes was obtained from the *B. napus* genome annotation results and mapped to the corresponding chromosomes. The scale bar indicates the genome size of *B. napus*.

**Figure 4:**
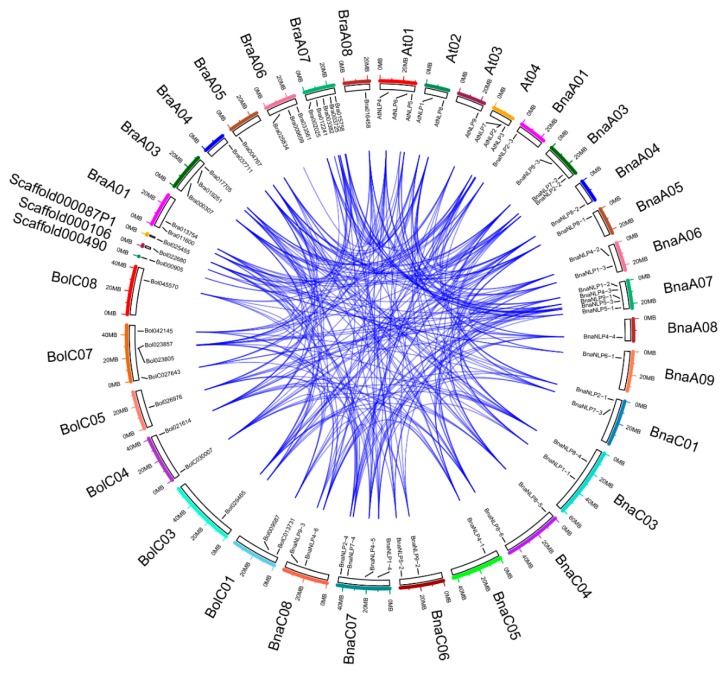
Syntenic relationships of *NLP* genes in *A. thaliana*, *B. napus*, *B. rapa*, and *B. oleracea*.

Chromosome location analysis revealed that 31 *BnaNLP* genes are located on 16 chromosomes in *B. napus*, with 16 genes in the A subgenome and 15 in the C subgenome (Figure 3, Table S2). Four *BnaNLP* genes were identified on chromosomes A06 and A07, and three were found on chromosomes A03 and C07. Two *BnaNLP* genes each were found on chromosomes A01, C01, C03, C04 and C06, and only one *BnaNLP* gene was found on chromosomes A04, A05, A08, A09, C05 and C08. No *BnaNLP* genes were found on chromosomes A01, A010, C01 and C09. In addition, all *BnaNLP* genes share syntenic relationships with *NLP* members in *A. thaliana*, *B. rapa*, and *B. oleracea* (Figure 4), and no tandemly duplicated *BnaNLP* family genes were identified.

To explore the selective pressure on *BnaNLP* genes, we calculated the non-synonymous/synonymous mutation ratio (Ka/Ks); Ka/Ks > 1 indicates positive selection, Ka/Ks = 1 indicates neutral selection, and Ka/Ks < 1 indicates purifying selection [30]. The Ka/Ks ratio for all *BnaNLP* genes was <1, ranging from 0.0842 (*BnaNLP4-1*) to 0.5926 (*BnaNLP8-4*), indicating that these genes are functionally conserved (Table 2). A comparison of the Ka/Ks ratios of *BnaNLP* genes between the A and C subgenomes showed that the average Ka/Ks ratio was higher in the C subgenome (0.1901) than in the A subgenome (0.1728), suggesting that *BnaNLP* genes in the C subgenome experienced higher selection pressure during the evolutionary history of *B. napus*. A comparison of Ka/Ks ratios among the three groups indicated that the average Ka/Ks ratio in group III was higher than that in groups I and II and that *BnaNLP* genes in group II had the lowest average Ka/Ks ratio among the three groups (Table 2). Unlike the *BnaNLP8* subfamily, most homologous members in the remaining subfamilies possessed similar Ka/Ks ratios, suggesting that the homologous genes in the *BnaNLP8* subfamily might have been subjected to different evolutionary pressures after the whole genome triplication (WGT) event in *B. napus*.

### 2.4. Gene Structure and Conserved Motif Analyses

To compare the exon/intron structures of *AtNLPs* and *BnaNLP* genes, we aligned the coding sequences with their corresponding genomic sequences. Similar gene structures were not only observed in homologous genes in *B. napus*, but they were also found within *NLP* homologs in *B. napus* and *A. thaliana* (Figure 1B). Most *BnaNLP* members in groups I and II contain four exons and three introns, while *BnaNLP2-1/4-6/4-2* genes contain five exons and four introns. Most members in group III contain at least six exons and five introns, except for *BnaNLP8-3* (five exons and four introns) and *BnaNLP8-6* and *BnaNLP9* (seven exons and six introns each). The similar gene structures observed in the same *BnaNLP* subfamilies are consistent with their phylogenetic relationships.

To better understand the structural diversity of BnaNLP proteins, we identified 15 putative motifs in the proteins using the MEME/MAST program (Figure 1C,D). Motifs 1/2/3/4/5/7/9 were observed in all 31 BnaNLP proteins, while motifs 6/10/12 were absent in BnaNLP4-4 protein, and motifs 8/11 were not detected in BnaNLP3-1 protein. While motifs 14/15 were detected in most BnaNLP proteins, motif 14 was absent from the BnaNLP8 and BnaNLP9 subfamilies and motif 15 was absent from BnaNLP4-3/4-6 proteins. Motif 13 was only detected in the BnaNLP7 subfamily.

Sequence alignment showed that motif 1 is the conserved GAF domain. InterProScan annotation showed that motif 2 is the RWP-RK domain, which plays a key role in N-mediated control of development. Motif 6 is the PB1 domain, comprising approximately 80 amino acid residues. BnaNLP proteins in the same group share similar motifs, suggesting that similar BnaNLP members share conserved biological functions.

### 2.5. Prediction of TF Binding Sites in the BnaNLP Promoters

TF binding sites (TFBSs) are short, specific DNA sequences that bind with TFs to regulate the transcription of genes. TFBS identification is a key step in understanding transcriptional regulation mechanisms and establishing transcriptional regulatory networks. To further understand the transcriptional regulation and potential functions of *BnaNLPs* genes, we screened for TFBSs in the 2000-bp upstream promoter sequences of these genes using PlantPan2.0 [31]. Sixty-three types of TFBSs were detected in the promoters of *BnaNLP* genes, involving 43 TF families (Table S3). TFBSs for 29 TF families were observed in all of the *BnaNLP* promoters, such as binding sites for AP2, GATA, NAC, and MADS-box TFs. In addition, *BnaNLP9-2* might be associated with physiological processes, such as plant resistance and aging, and thus its promoter contained more binding sites for the WRKY TFs compared with other family members (Table S3).

### 2.6. Three-Dimensional Structure Prediction

**Figure 5:**
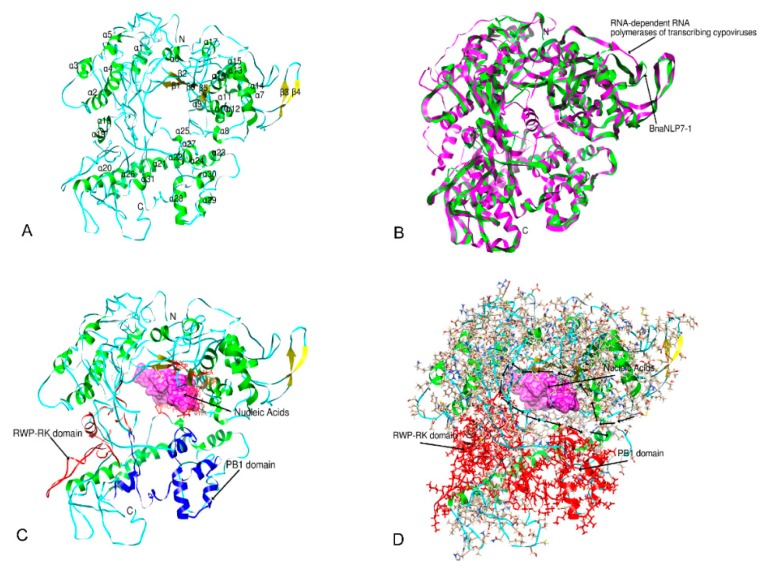
Predicted 3D structures of BnaNLP7-1 protein. The models were obtained using I-TASSER. tRNA-dependent RNA polymerases of transcribing cypoviruses (PDB ID: 3JA4) were used as the template for 3D structure predication. (**A**) 3D structure of BnaNLP7-1 protein; (**B**) template model of 3JA4 and 3D structure of BnaNLP7-1 protein; (**C**,**D**) binding between the BnaNLP7-1 dock and its nucleic acid substrate (PBD: 2r7rA); (**C**) the conserved PWP-PK domain and PB1 motifs are shown on the 3D structure in red and blue, respectively. The surface of the nucleic acid substrate is shown in purple. (**D**) The conserved PWP-PK domain and PB1 motifs are shown in red on the 3D structure. *α*-helices, *β*-strands, and random coils are indicated in green, yellow, and navy blue, respectively. All images were generated using Chimera 1.2.

We predicted the three-dimensional structure of BnaNLP7-1 protein, a representative BnaNLP proteins, using I-TASSER [32]. The best template used for the 3-D structure prediction was the RNA-dependent RNA polymerases of transcribing cypoviruses [33]. The sequence identity between BnaNLP7-1 and the 3JA4 template was 0.18 across the whole template. The coverage of the threading alignment (equal to the number of aligned residues divided by the length of the query protein) was 0.96. Alignment with a normalized Z-score > 1 indicates good alignment and vice versa. The normalized Z-score of the threading alignments was 2.83, suggesting that good alignment was obtained in our prediction. The modeled structure for BnaNLP7-1 has 31 *α*-helices and five *β*-strands (Figure 5A). The RWP-RK domain contains two *α*-helices (*α*18 and *α*19), while the PB1 domain contains five *α*-helices (*α*27, *α*28, *α*29, *α*30, and *α*31). The two conserved domains also possess many loops and lack *β*-strands.

NLP proteins are plant-specific TFs that play key roles in regulating Nitrogen responses. To gain deeper insight into their biological function, COFACTOR and COACH [33] were used to predict the binding activities of the BnaNLP proteins with the promoters of downstream genes. The 2r7rA ligand (PBD: 2r7rA) [34] was used to bind BnaNLP7-1 protein as the nucleic acid substrate. The docking results showed that Gln320, Ala322, Leu323, Gln343, Thr344, Trp345, Gly369, Asp381, Ala383, Ala499, Ser500, Gly501, Phe767, and Pro768 are involved in ligand binding (Figure 5C). Interestingly, the binding of BnaNLP7-1 and a nitrogen deficiency-responsive cis-element form a ring that associates with polymerase II transcription (Figure 5D). No binding ability was detected for two conserved domains (RWP-RK and PB1) due to the lack of a binding site in their sequences, suggesting that those two conserved domains not function in ligand binding.

### 2.7. Organ-Specific Expression Patterns of BnaNLP Genes

**Figure 6:**
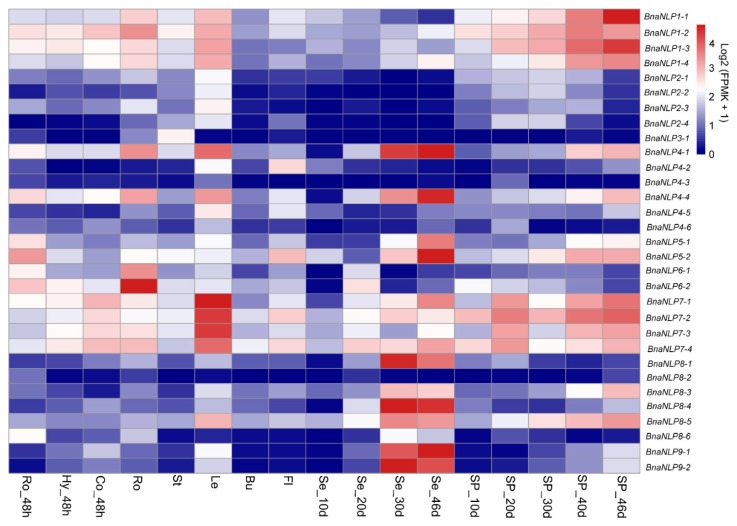
Organ-specific expression profiles of *BnaNLP* genes. Organ-specific expression patterns of *BnaNLP* genes were obtained based on the RNA-seq data. The color bar at the upper right side of the figure represents Log_2_ (FPKM + 1), with blue representing little or no expression. Ro_48h: Roots collected at 48 h after germination; Hy_48: Hypocotyls harvested at 48 h after germination; Co_48h: cotyledons collected at 48 h after germination; Ro, St, Le, Bu and Fl: Roots, stems, leaves, buds and flowers harvested at the initial blooming stage; Se_10d, Se_20d, Se_40d and Se_46d: Seeds harvested at 10, 20, 40, and 46 days after flowering; SP_10d, SP_20d, SP_30d, SP_40d, and SP_46d: Silique pericarps harvested at 10, 20, 30, 40, and 46 days after flowering.

To investigate the organ-specific expression profiles of the *BnaNLP* genes, we subjected 17 types of organ to RNA-seq. Calculation of the expression levels of the *BnaNLP* genes revealed diverse expression patterns in different subfamilies (Figure 6). Four *BnaNLP1* subfamily members were highly expressed in silique pericarps (SP) at 30 days after flowering (DAF), especially *BnaNLP1-1* and *BnaNLP1-3*. Four *BnaNLP2* subfamily members and *BnaNLP3* were expressed at extremely low levels in all organs examined. *BnaNLP4-1*, *BnaNLP4-4*, *BnaNLP8-1*, *BnaNLP8-4*, *BnaNLP9-1* and *BnaNLP9-2* were expressed at high levels in seeds at 40 and 3 DAF. Two *BnaNLP5* genes were strongly expressed only in seeds at 46 DAF. Unlike the abovementioned subfamilies, two *BnaNLP6* subfamily members were mainly expressed in roots, and four *BnaNLP7* subfamily members were highly expressed in leaves. These results indicate that most *BnaNLP* genes were expressed at different levels in different organs and were preferentially expressed in specific organs.

### 2.8. Expression Patterns of BnaNLP Genes under Nitrogen Deficiency

**Figure 7:**
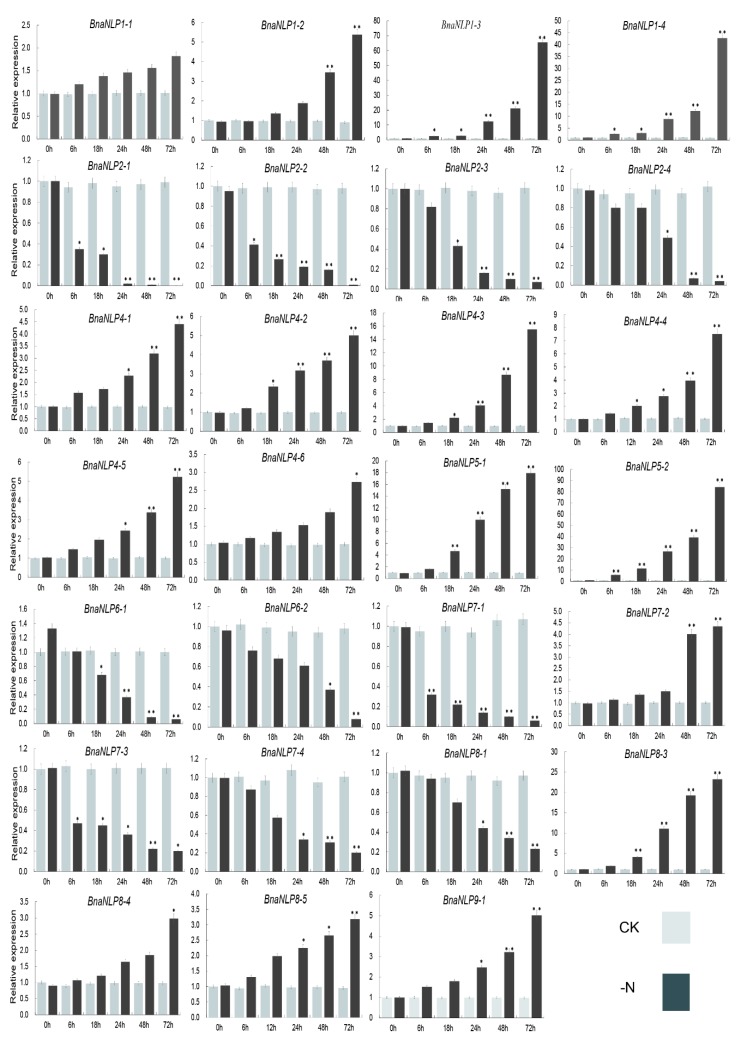
Expression profiles of *BnaNLP* genes in SRs under normal and nitrogen deficient conditions. Relative expression levels of *BnaNLP* genes were determined by RT-qPCR. SRs were collected under normal growth and nitrogen deficient conditions at 0, 6, 12, 24, 48, and 72 h after treatment. * represents the significant level, ** represents the extremely significant level.

To investigate the expression patterns of *BnaNLP* genes under nitrogen deficiency, seedling leaves (SLs) and (seedling roots) SRs were harvested at various time points (0 to 72 h) after culturing in Hoagland nutrient solution with or without Nitrogen. In SLs, *BnaNLP8-3*, *BnaNLP8-4*, *BnaNLP8-5*, *BnaNLP9-1*, *BnaNLP1*, *BnaNLP4*, and *BnaNLP5* subfamily members were upregulated under nitrogen deficiency treatment, while other members were downregulated (Figure 7). Most *BnaNLPs* were significantly upregulated or downregulated after 48–72 h of nitrogen deficient treatment, except for *BnaNLP1-1* and *BnaNLP8-4*. In addition, a few genes were upregulated immediately after treatment and subsequently downregulated after 6 to 18 h, such as *BnaNLP6-1* and *BnaNLP8-1*.

**Figure 8:**
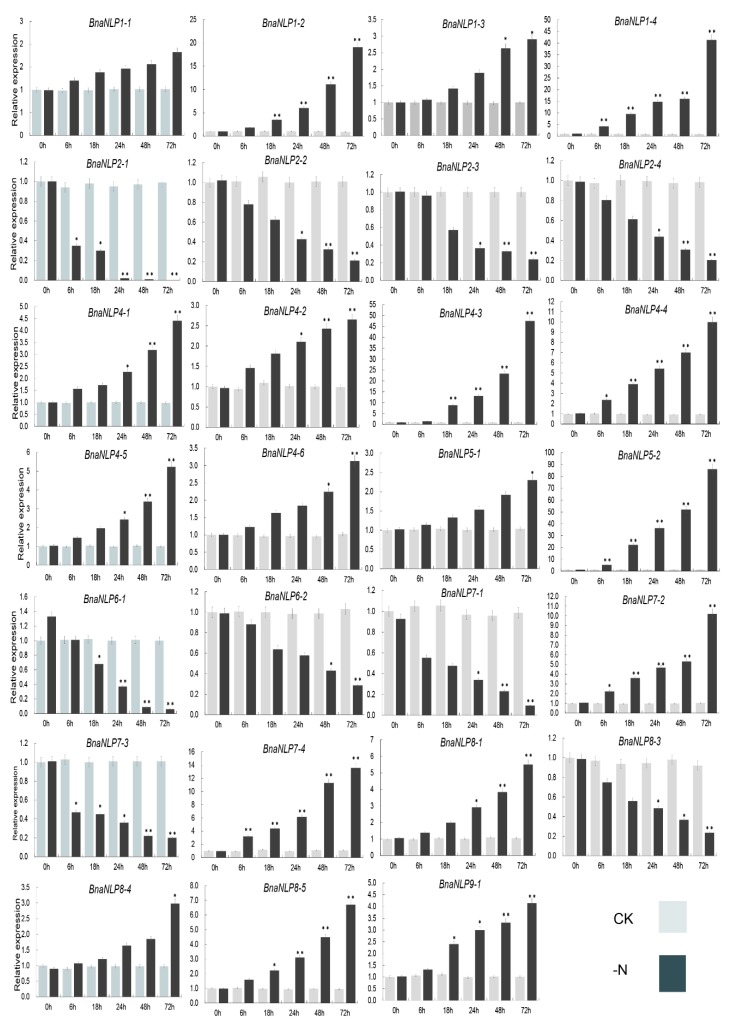
Expression profiles of *BnaNLP* genes in SLs under normal and nitrogen deficient conditions. Relative expression levels of *BnaNLP* genes were determined by RT-qPCR. SRs were collected under normal growth and nitrogen deficient conditions at 0, 6, 12, 24, 48 and 72 h after treatments. * represents the significant level, ** represents the extremely significant level.

Interestingly, the responses of most *BnaNLP* genes in SRs to nitrogen deficiency treatment were highly similar to those in SLs (Figure 7 and Figure 8). *BnaNLP1-3* and *BnaNLP1-4* were the most sensitive genes to nitrogen deficiency treatment in SLs, whereas *BnaNLP4-3* was most sensitive to this treatment in SRs (Figure 7 and Figure 8). Among the four *BnaNLP7* subfamily members, three (*BnaNLP7-1*, *BnaNLP7-3*, and *BnaNLP7-4*) were downregulated after treatment in SLs, while *BnaNLP7-2* was upregulated (Figure 7). *BnaNLP7-1/3* were downregulated and BnaNLP7-2/4 were upregulated after treatment in SRs. In general, members of the same subfamily often shared similar expression patterns, except for members of the *BnaNLP7/8* subfamilies, consistent with their phylogenetic relationships.

## 3. Discussion

### 3.1. Characterization of the NLP Gene Family in B. napus

Previous studies have revealed nine *NLP* family genes in *Arabidopsis* [8] and maize [17], six in rice [14], and five in sorghum [10]. In this study, we identified 31 *BnaNLP* family genes in *B. napus*. The *BnaNLP* gene family is larger than the *NLP* gene family in other plant species, because *B. napus* experienced an extra WGT and merging events [35]. Interestingly, *NLP* family genes have showed long protein residues and slightly lower pI values in several species [10,14,20,29]. Similar results were also detected in *BnaNLP* genes (Table 1), which illustrated that this TFs prefer to be active in acidic conditions. In *maize*, most *ZmNLP* genes possessed four to six exons, with the exception of *ZmNLP1* which contained seven exons [20]. Similarly, for *NLP* genes in *Populus trichocarpa*, most *NLP* also contained four to six exons, except for *PtrNLP1* (two exons) and *PtrNLP4* (seven exons) [29]. In this study, different gene structures were found among *BnaNLP* family members, and group I and II members had fewer exons (four to five) than group III members (five to six, *BnaNLP8-6* and BnaNLP9 possessed seven exons), implying structural diversification among the *BnaNLP* subfamilies. NLP proteins contain two conserved domains, RWP-RK and PB1. All BnaNLP proteins identified in this study contain two domains, except for BnaNLP4-4, which contains only the RWP-RK domain. Sequence analysis of BnaNLP4-4 indicated that more than 50 amino acids (including the PB1 domain) are absent in its C-terminus, which was caused by sequence fragmentation in the evolution or sequencing error in the *B. napus* genome. The RWP-RK domain contains a helix-turn-helix motif and an amphipathic leucine zipper, which might be involved in DNA binding [10,11]; further studies showed that its activity is unrelated to nitrate signaling, whereas the N-terminal regions of NLPs function as a transcriptional activation domain to mediate this signaling [22]. However, our docking results suggested that this domain may not be involved in the binding of NLP protein with nucleic acids. The PB1 domain contains two *α*-helices and a mixed five-stranded *β*-sheet, which might play a key role in its protein-binding ability [15]. We found that the PB1 domain acts like a switch, which may involve controlling the initiation or stopping the binding process. Hence, the biological function of the PB1 domain in the protein binding process deserved further elucidation.

Previous studies suggested that the structurally characterized GAF domains bind with low-molecular-weight ligands, including cGMP, 2-oxoglutarate, nitric oxide and nitrate, or serve as homodimerization modules associated with gene regulation in organisms ranging from bacteria to higher plants [3,36,37]. Due to the limitation of sequence identity between BnaNLP7-1 and the template, the binding site for GAF domain was not identified in our docking results. But we found the conserved sensing and signaling GAF domain in all BnaNLP proteins. Although legumes have specifically evolved as the NIN gene with functional responsibility for symbiotic nitrogen fixation, the presence of these three domains (GAF, RWP-RK, PB1) allows the NLPs to function in various aspects of nitrogen responses in non-nitrogen fixating plants, including nitrogen sensing, transcriptional modulation, and signal transduction [20]. In this study, all BnaNLP proteins possessed the three domains (besides BnaNLP4-4), reflecting the pivotal roles of BnaNLP proteins in rapid response and adaptation to nitrogen deficiency in *B. napus*.

### 3.2. Phylogenetic Relationship and Duplication Analysis of BnaNLPs

At least three ancient copies of *NLP* genes are thought to have existed in the ancestor of monocots and dicots [10], and two *NLP* genes have experienced one round of duplication in *Arabidopsis*, whereas the third gene has undergone several rounds of duplication since the divergence of eudicots and monocots. Our phylogenetic analysis of *NLP* genes in nine plant species provided evidence for the hypothesis that the ancestor of monocots and dicots contained at least three *NLP* gene family members (Figure 2), since there is at least one *NLP* gene in each of three *NLP* groups in *A. trichopoda*. Our results suggest that the ancestor of *NLPs* in most plant species has experienced one round of duplication, while the ancestor of *NLPs* in group III may have been duplicated more than once in the Brassicaceae lineages, perhaps due to the WGT event that have occurred in Brassicaceae species.

In general, *B. rapa* and *B. oleracea* contain three syntenic copies of each *A. thaliana* gene [28,29] and *B. napus* contains six, since it was generated from *B. rapa* and *B. oleracea* [35]. In this study, 0–2 copies of each *NLP* gene were identified in *B. rapa* and *B. oleracea* and 1–6 copies were identified in *B. napus*, perhaps due to genome shrinkage or gene loss after WGT. We detected 31 syntenic copies of *BnaNLP* genes and 16 syntenic copies of *BraNLP* genes in ancient WGT blocks, suggesting that the *NLP* gene family in the A subgenome of *Brassica* crops is highly conserved. However, 14 syntenic gene copies were found in ancient WGT blocks in *B. oleracea*, only eight of which contained both the RWP-RK and PBl domains, perhaps due to assembly errors of *B. oleracea* genome.

### 3.3. Expression Profiling and Response of Genes to Nitrogen Deficient Conditions

The expression patterns of *NLP* genes in different organs have been investigated in several plants. In rice and Arabidopsis, *NLPs* are expressed in almost all organs [14]. *AtNLP8* and *AtNLP9* are preferentially expressed in senescent leaves and seeds and are expressed at moderate or very low levels in other organs [14]. In rice, *OsNLP1* and *OsNLP3* are highly expressed in source organs, representing the most abundant *OsNLP* transcripts [14]. *NLPs* in *P. trichocarpa* are highly expressed in leaves, roots, male catkins, xylem, seeds, and female catkins [29]. In this study, we found that most *BnaNLP* genes were expressed in leaves, while only the four *BnaNLP7* subfamily members were highly expressed in leaves (Figure 6). Similar to previous reports, the accumulations of *BnaNLP* genes in leaves might also be used for storing nitrogen to coordinate leaf expansion and photosynthetic capacity, and nitrogen supply can improve their storage to promote leaf growth and biomass [38]. *BnaNLP1* genes were highly expressed in silique pericarps, the main source organ during the podding stage in *B. napus*, suggesting that *BnaNLP1* subfamily members may be involved in nitrogen storage or supply mainly in silique pericarps. In addition, six *NLPs* (*BnaNLP4-1*, *BnaNLP4-4*, *BnaNLP8-1*, *BnaNLP8-4*, *BnaNLP9-1*, and *BnaNLP9-2*) were strongly expressed in seeds at 40 and 46 DAF, suggesting that these genes may function in nitrogen assimilation during seed maturation. *BnaNLP6* subfamily members were primarily expressed in roots, which is similar to the expression patterns of *NLP* genes in maize [20]. Different from nitrogen-fixing plants, the sources of nitrogen in *Brassica* species are mainly based on the absorption of nitrate nitrogen in soil though roots [2,39]; the strong expression of *BnaNLP6* subfamily in root may reflect the important roles of *BnaNLP6* in nitrogen absorption in roots of *B. napus*. We found that members of the same subfamily share similar expression profiles, including *BnaNLP1*, *BnaNLP2*, *BnaNLP5*, *BnaNLP6*, *BnaNLP7* and *BnaNLP9* subfamilies, suggesting basic functional conservation within each subfamily [14]. However, members of the *BnaNLP4* or *BnaNLP8* subfamily showed less similar expression profiles and some of them are not expressed at any organs, illustrating that nonfunctionalization or subfunctionalization occurred in the two *BnaNLP* subfamilies.

Nitrogen deficiency treatment can trigger rapid, extensive transcriptional changes of genes involved in a wide range of cellular and physiological processes [40,41]. In the current study, most *BnaNLP* genes were up- or down-regulated under nitrogen deficiency treatment in both SLs and SRs, due to the specific functions in nitrogen remobilization during nitrogen deficiency [42]. These upregulated *BnaNLP* genes may play key roles in the distribution and remobilization of nitrogen in response to the short-time demand for nitrogen during nitrogen deficiency. Interestingly, the induction of *BnaNLP* genes was slower than that observed for the homologs in maize, which were up/downregulated within 2 h of nitrate treatment [20], pointing to different nitrogen utilization mechanisms between *B. napus* and maize.

In *Arabidopsis*, *AtNLP7* regulates nitrate and nitrogen starvation responses [9] and was identified in a genetic screen for regulators of the primary nitrate response [2]. *AtNLP7* binds to the promoters of 851 genes in response to nitrate signals, binding preferentially to the transcriptional start sites of target genes. Moreover, the accumulation of *AtNLP7* in the nucleus occurs independently of transcriptional regulation, and inhibiting nuclear export mimics the nitrate signal [24]. In this study, we identified four *BnaNLP7* subfamily members. Of these, three (*BnaNLP7-1/7-3/P7-4*) were downregulated and only the *BnaNLP7-2* expression was upregulated in SLs after nitrogen deficiency treatment (Figure 7). However, *BnaNLP7-1/7-3* were downregulated and *BnaNLP7-2/7-4* were upregulated in SRs after nitrogen deficiency treatment, suggesting that *BnaNLP7* subfamily members have experienced subfunctionalization during the polyploidy evolution of *B. napus*. The three different expression patterns in *BnaNLP7* subfamily (upregulated in both SLs and SRs (*BnaNLP7-2*), downregulated in both SLs and SRs (*BnaNLP7-1/7-3*) and the exhibition of opposite regulated patterns in SLs vs. SRs (*BnaNLP7-4*)) point to the presence of at least three types of molecular mechanisms that function in response to nitrogen deficiency. Based on functional studies of *AtNLP7*, it could be speculated that there might be at least three groups of genes which are regulated by different *BnaNLP7* subfamily members. Under the condition of nitrogen deficiency, upstream nitrogen signaling pathway genes activate different *BnaNLP7* subfamily members in different organs, then the post-translationally activated *BnaNLP7* regulate the expression of nitrate-inducible assimilation and downstream regulatory genes through interaction with NREs to improve the nitrogen absorption in roots and nitrogen assimilation in whole *B. napus* plants.

In summary, we found that *BnaNLP* genes have various expression patterns and most of them are extremely sensitive to nitrogen deficiency. This provides insights into the biological functions of *BnaNLP* genes in response to nitrogen deficiency.

## 4. Materials and Methods

### 4.1. Plant Materials and Treatments

Seeds of *Brassica napus* cultivar ZS11 were germinated in plant growth chambers (under a 16 h photoperiod at 25/18 °C day/night, 60% humidity, 250 µmoles/m^2^/s; PGC Flex, Conviron, MB, Canada) and transplanted into a field in Chongqing, China. To detect the expression patterns of *BnaNLP* family members in different organs, root (Ro), hypocotyl (Hy), and cotyledon (Co) organs were collected at 48 h after seed germination. Ro, stem (St), mature leaf (Le), bud (Bu), and flower (Fl) organs were harvested at the initial blooming stage, while seeds (Se) were harvested at 10, 20, 40, and 46 DAF, and silique pericarps (SP) were harvested at 10, 21, 30, 40, and 46 DAF in the field. Two biological replicates per sample were collected for RNA sequencing (RNA-seq) analysis.

To investigate the expression patterns of *BnaNLP* genes under nitrogen deficiency, *B. napus* seedlings were cultivated in pots in full-strength Hoagland solution without sand as a substrate in a plant growth chamber with a thermo-photoperiod of 25 °C for 16 h/18 °C for 8 h (light/dark) [43]. One-month-old seedlings were transferred into new full-strength Hoagland solution or modified N-deficient Hoagland nutrient solution for treatment. All nutrition solutions were renewed every 3 days. Three biological replicates of SLs and SRs were harvested after 0, 6, 18, 24, 48, and 72 h of treatment. All samples were immediately frozen in liquid nitrogen and stored at −80 °C.

### 4.2. Identification of NLP Family Genes in Plants

Genomic, coding sequences, and protein sequences from *A. thaliana*, *B. napus*, *B. rapa*, and *B. oleracea* were downloaded from the *Arabidopsis* Information Resource (TAIR, http://www.arabidopsis.org) and the *Brassica* Database (BRAD, http://brassicadb.org/brad). Sequences from *A. trichopoda*, rice, maize, sorghum, and grape were obtained from Phytozome 12.0 (www. phytozome.net).

To identify *NLP* genes in these species, nine NLP protein sequences from *Arabidopsis* were used as queries in a reciprocal Basic Local Alignment Search Tool Protein (BLASTP) analysis [44,45] using the threshold and minimum alignment coverage parameters described previously [46]. All NLP protein sequences were further analyzed using PfamScan (http://www.ebi.ac.uk/Tools/pfa/pfamscan/) to confirm the presence of two NLP-related domains, RWP-RK (PF02042) and PBl (PF00564), with an *e*-value cut-off of 1 × 10^−5^.

### 4.3. Phylogenetic Analysis of the NLP Family in B. napus

Multiple sequence alignments of *NLP* coding sequences between *B. napus* and *A. thaliana* and of protein sequences in the nine plant species (*A. thaliana*, *B. napus*, *B. rapa*, *B. oleracea*, rice, maize, sorghum, grape and *A. trichopoda*) mentioned above were conducted using ClustalW2 with default parameters (http://www.genome.jp/tools-bin/clustalw). The phylogenetic trees were generated using the Molecular Evolutionary Genetics Analysis (MEGA) 7 program (Tokyo Metropolitan University, Tokyo, Japan) [47] with the NJ method, the *p*-distance + G substitution model, 1000 bootstrap replications, and conserved sequences with a coverage of 70%. The phylogenetic trees were visualized using FigTree v1.4.0 (http://tree.bio.ed.ac.uk/software/figtree/). The coding sequence alignments were imported into KaKs_calculator2.0 (Chinese Academy of Sciences, Beijing, China) to calculate the synonymous mutation rate (Ks) and non-synonymous mutation rate (Ka) using the NG method [22].

### 4.4. Chromosomal Locations and Protein Property Analysis

Chromosomal locations of the *BnaNLP* genes and the orthologous relationships between *A. thaliana* and *Brassica* species were determined based on the results of reciprocal BLASTP analysis and the general feature format (GFF) files downloaded from the Brassica database (http://brassicadb.org/brad). The chromosome distribution was plotted with MapChart2.0 (https://mapchart.net/) [48].

The molecular weight (MW) and isoelectric point (pI) of each BnaNLP protein were predicted using the ExPASy server (http://expasy.org). The transmembrane transport peptides were predicted using Tmpred [49], and signal peptides were predicted using SignalP4.1 (http://www.cbs.dtu.dk/services/SignalP/) [50]. Subcellular localization of BnaNLP was predicted using an extensive high-performance subcellular protein localization prediction system MultiLoc2.0 server (http://abi.inf.uni-tuebingen.de/Services/MultiLoc2/) by incorporating phylogenetic profiles and Gene Ontology terms [51], with the predictive method MultiLoc2-HighRes (Plant), 10 Localization. Gene Structure Display Server (GSDS 2.0, http://gsds.cbi.pku.edu.cn) was used to display the exon/intron structures of *BnaNLP* genes.

### 4.5. Motif Identification and TF Binding Site Analysis

Conserved motifs in the proteins were identified using the Expectation Maximization for Motif Elucidation program (MEME v4.12.0, http://meme-suite.org) with the following parameter settings: The maximum number of motifs was 15, and the optimum width was set from 6 to 200. Only motifs with an *e*-value of 1e-10 were retained for further analysis. The promoter sequences (2-kb upstream region) of the *BnaNLP* genes were obtained from the *B. napus* genome (http://www.genoscope.cns.fr/brassicanapus). PlantPAN2.0 (http://plantpan2.itps.ncku.edu.tw) was used to analyze TF binding sites (TFBSs).

### 4.6. Three-Dimensional Structure Prediction of BnaNLP7-1

The three-dimensional (3D) structure of BnaNLP7-1 protein was predicted using the I-TASSER program (https://zhanglab.ccmb.med.umich.edu/I-TASSER/) [32]. To identify the best structural template for BnaNLP7-1 in the Protein Data Bank (PDB) database [52], the query sequence was subjected to multiple rounds of threading using LOMETS [53]. RNA-dependent RNA polymerases of transcribing cypoviruses (PDB ID: 3JA4) [33] were the best structural template for our queried protein. The newly generated 3D model was aligned to the template using TM-align, and COFACTOR was used to predict the binding sites of ligands to the protein structure [54,55]. Chimera 1.2 (https://www.cgl.ucsf.edu/chimera/) was used to examine and visualize the newly generated 3D model of BnaNLP7-1.

### 4.7. RNA Isolation, RNA-Sequencing, and Quantitative Reverse-Transcription PCR

Total RNA was extracted from the organ using an RNA Extraction Kit (Tiangen, Beijing, China), and cDNA was synthesized from 1 µg of total RNA using M-MLV transcriptase (TaKaRa Biotechnology, Dalian, China) according to the manufacturer’s instructions.

To determine the organ-specific expression profiles of *BnaNLP* genes, publicly available *B. napus* RNA-seq data (BioProject ID PRJNA358784) were used to quantify the expression levels of the *B. napus* genes in different organs as fragments per kilobase of exon per million reads mapped (FPKM) values using Cufflinks with default parameters. Two independent biological replicates were analyzed per sample. The expression values of the 31 *BnaNLP* genes were extracted from the RNA-seq results and normalized by Log2 (FPKM + 1).

Reverse transcription quantitative PCR (RT-qPCR) was performed on a Bio-Rad CFX96 Real Time System (USA) according to a previously described method [20]. Gene-specific primer sequences for the *BnaNLP* genes were obtained from the qPrimerDB database [56]. (Table S1). Each reaction was carried out in biological triplicate in a reaction volume of 20 µL containing 1.6 µL of gene-specific primers (1.0 µM), 2.0 µL of cDNA, 10 µL of SYBR green, 0.2 µL of ROX Reference Dye II, and 6 µL of sterile distilled water. The PCR program was as follows: 95 °C for 3 min; 45 cycles of 10 s at 95 °C and 30 s at 60 °C; and then melt curve 65 °C to 95 °C, increment 0.5 °C for 5 s. Melting curves were generated to estimate the specificity of these reactions. Relative expression levels were calculated using the 2^−ΔΔCt^ method, with *BnaACT7* and *BnaUBC21* used as internal controls [57].

## Figures and Tables

**Table 1:** List of *BnaNLP* genes identified in *B. napus*.

Gene Name in *B. napus*	Gene ID in *B. napus*	Gene ID in *A. thaliana*	Chromosome	Position (bp)	Number of Introns	Protein Length (aa)	Protein Molecular Weight (kDa)	Protein Isoelectric Point (pI)	Protein Subcellular Localization
*BnaNLP1-1*	*BnaC03g47510D*	*AT2G17150*	C03	32,561,336~32,565,236	3	870	95.78	5.1	Nucleus
*BnaNLP1-2*	*BnaA07g03130D*	*AT2G17150*	A07	2,770,119~2,774,330	3	895	99.32	4.98	Nucleus
*BnaNLP1-3*	*BnaA06g25930D*	*AT2G17150*	A06	17,936,012~17,942,485	3	872	96.02	5.18	Nucleus
*BnaNLP1-4*	*BnaC07g06170D*	*AT2G17150*	C07	9,960,829~9,965,297	3	896	99.33	4.98	Nucleus
*BnaNLP2-1*	*BnaA01g02150D*	*AT4G35270*	A01	1,061,127~1,065,935	4	889	98.91	6.87	Nucleus
*BnaNLP2-2*	*BnaA03g53210D*	*AT4G35270*	A03	27,917,856~27,921,412	3	897	100.07	6.67	Nucleus
*BnaNLP2-3*	*BnaC01g03280D*	*AT4G35270*	C01	1,694,787~1,699,810	3	966	107.19	5.92	Nucleus
*BnaNLP2-4*	*BnaC07g45500D*	*AT4G35270*	C07	43,543,401~43,546,699	3	901	100.71	7.04	Chloroplast
*BnaNLP3-1*	*BnaA06g40940D*	*AT4G38340*	A06	2,255,358~2,256,385	3	681	76.37	7.31	Nucleus
*BnaNLP4-1*	*BnaA06g14540D*	*AT1G20640*	A06	7,867,109~7,871,401	3	814	91.05	5.59	Nucleus
*BnaNLP4-2*	*BnaA07g11340D*	*AT1G20640*	A07	10,591,730~10,594,794	3	775	86.88	5.36	Nucleus
*BnaNLP4-3*	*BnaA08g21720D*	*AT1G20640*	A08	15,991,108~15,994,588	4	764	85.19	5.61	Nucleus
*BnaNLP4-4*	*BnaC05g16000D*	*AT1G20640*	C05	9,745,808~9,749,062	3	703	77.85	5.84	Nucleus
*BnaNLP4-5*	*BnaC07g15260D*	*AT1G20640*	C07	21,212,376~21,215,440	3	769	86.07	5.47	Nucleus
*BnaNLP4-6*	*BnaC08g19370D*	*AT1G20640*	C08	22,293,545~22,296,521	4	761	85.02	5.75	Nucleus
*BnaNLP5-1*	*BnaA07g32630D*	*AT1G76350*	A07	22,548,833~22,552,146	3	802	89.64	5.89	Nucleus
*BnaNLP5-2*	*BnaC06g37080D*	*AT1G76350*	C06	35,298,227~35,301,829	3	802	89.61	5.94	Nucleus
*BnaNLP6-1*	*BnaA09g12180D*	*AT1G64530*	A09	6,394,926~6,398,454	5	822	91.48	5.56	Nucleus
*BnaNLP6-2*	*BnaCnng38990D*	*AT1G64530*	Unknown		5	822	91.56	5.74	Nucleus
*BnaNLP7-1*	*BnaA01g35090D*	*AT4G24020*	A01	386,973~387,241	5	934	102.95	5.5	Nucleus
*BnaNLP7-2*	*BnaA03g46410D*	*AT4G24020*	A03	23,822,691~23,826,304	5	934	102.68	5.6	Nucleus
*BnaNLP7-3*	*BnaC01g15850D*	*AT4G24020*	C01	10,891,090~10,895,162	5	978	108.05	5.96	Nucleus
*BnaNLP7-4*	*BnaC07g38670D*	*AT4G24020*	C07	39,940,917~39,945,105	5	937	103.06	5.61	Nucleus
*BnaNLP8-1*	*BnaA03g20260D*	*AT2G43500*	A03	9,638,886~9,642,606	5	895	98.71	5.41	Nucleus
*BnaNLP8-2*	*BnaA04g25110D*	*AT2G43500*	A04	18,201,925~18,205,255	5	798	88.35	5.94	Nucleus
*BnaNLP8-3*	*BnaA05g03380D*	*AT2G43500*	A05	1,878,196~1,880,941	4	809	89.36	5.94	Nucleus
*BnaNLP8-4*	*BnaC03g24230D*	*AT2G43500*	C03	13,588,155~13,591,741	5	897	99.00	5.42	Nucleus
*BnaNLP8-5*	*BnaC04g02980D*	*AT2G43500*	C04	2,115,457~2,118,921	5	799	88.08	5.92	Nucleus
*BnaNLP8-6*	*BnaC04g48980D*	*AT2G43500*	C04	47,362,522~47,365,605	6	811	90.07	5.94	Nucleus
*BnaNLP9-1*	*BnaA07g18430D*	*AT3G59580*	A07	15,037,510~15,041,748	6	852	94.09	5.47	Nucleus
*BnaNLP9-2*	*BnaC06g17440D*	*AT3G59580*	C06	20,075,721~20,079,938	6	851	94.15	5.58	Nucleus

**Table 2:** Non-synonymous and synonymous nucleotide substitution rates between *AtNLPs* and the corresponding orthologs in *B. napus*.

Group	Gene ID in *A. thaliana*	Gene ID in *B. napus*	Model	Ka	Ks	Ka/Ks	Average Ka/Ks
Group I	*AT2G17150*	*BnaNLP1-1*	NG	0.095573	0.381817	0.250311	0.169549
	*AT2G17150*	*BnaNLP1-2*	NG	0.084925	0.375565	0.226126	
	*AT2G17150*	*BnaNLP1-3*	NG	0.094742	0.382276	0.247837	
	*AT2G17150*	*BnaNLP1-4*	NG	0.089774	0.376577	0.238395	
	*AT4G35270*	*BnaNLP2-1*	NG	0.064136	0.464096	0.138196	
	*AT4G35270*	*BnaNLP2-2*	NG	0.068387	0.409203	0.167123	
	*AT4G35270*	*BnaNLP2-3*	NG	0.063939	0.470639	0.135856	
	*AT4G35270*	*BnaNLP2-4*	NG	0.070692	0.431699	0.163754	
	*AT4G38340*	*BnaNLP3-1*	NG	0.123420	0.365014	0.338126	
	*AT1G20640*	*BnaNLP4-1*	NG	0.044027	0.522956	0.084189	
	*AT1G20640*	*BnaNLP4-2*	NG	0.057461	0.430702	0.133413	
	*AT1G20640*	*BnaNLP4-3*	NG	0.063875	0.37724	0.169322	
	*AT1G20640*	*BnaNLP4-4*	NG	0.056956	0.602642	0.094510	
	*AT1G20640*	*BnaNLP4-5*	NG	0.056344	0.418456	0.134646	
	*AT1G20640*	*BnaNLP4-6*	NG	0.063963	0.361807	0.176786	
	*AT1G76350*	*BnaNLP5-1*	NG	0.055669	0.60493	0.092026	
	*AT1G76350*	*BnaNLP5-2*	NG	0.057236	0.62409	0.091711	
Group II	*AT1G64530*	*BnaNLP6-1*	NG	0.069587	0.545178	0.127642	0.103387
	*AT1G64530*	*BnaNLP6-2*	NG	0.070799	0.519949	0.136166	
	*AT4G24020*	*BnaNLP7-1*	NG	0.027387	0.347158	0.07889	
	*AT4G24020*	*BnaNLP7-2*	NG	0.034582	0.363912	0.095029	
	*AT4G24020*	*BnaNLP7-3*	NG	0.029575	0.345875	0.085507	
	*AT4G24020*	*BnaNLP7-4*	NG	0.035223	0.3628	0.097086	
Group III	*AT2G43500*	*BnaNLP8-1*	NG	0.083688	0.393217	0.212829	0.264202
	*AT2G43500*	*BnaNLP8-2*	NG	0.105273	0.419328	0.251052	
	*AT2G43500*	*BnaNLP8-3*	NG	0.096503	0.434656	0.222021	
	*AT2G43500*	*BnaNLP8-4*	NG	2.395310	4.04226	0.592566	
	*AT2G43500*	*BnaNLP8-5*	NG	0.093713	0.471195	0.198883	
	*AT2G43500*	*BnaNLP8-6*	NG	0.107375	0.405135	0.265035	
	*AT3G59580*	*BnaNLP9-1*	NG	0.084630	0.469167	0.180383	
	*AT3G59580*	*BnaNLP9-2*	NG	0.089617	0.469558	0.190854	

## Supplementary Materials

Supplementary materials can be found at http://www.mdpi.com/1422-0067/19/8/2270/s1.
