# Supplementary material for: Genome-Wide Identification and Characterization of NODULE-INCEPTION-Like Protein (NLP) Family Genes in Brassica napus
Source: Int J Mol Sci. 2018 Aug 2;19(8):2270. doi: 10.3390/ijms19082270 (PMC6121332; doi:10.3390/ijms19082270)
Supplement: Supplementary file 1 [file ijms-19-02270-s001.zip › TableS1-2.docx]

**Supplementary tables**

Table S1 Primers used for RT-qPCR analysis of *BnaNLP* genes

| Gene name | Gene ID | Sequence of forward primer (5′-3′) | Sequence of reverse primer (5′-3′) |
| --- | --- | --- | --- |
| *BnaNLP1-1* | BnaC03g47510D | GCGTTTCAGCATAGATAACGTT | CCTCCCAGTTTGACTTGAAAAG |
| *BnaNLP1-2* | BnaA07g03130D | GTTTCAACAAGGTTCAACTCCA | CTTGTTATTCCATGGTGTCTGC |
| *BnaNLP1-3* | BnaA06g25930D | TCTGAGCAAGCAGAAAAGTTTC | CGTAAGAGTCTCTCCTTCACAG |
| *BnaNLP1-4* | BnaC07g06170D | TGCAGATTTTTGTGTATAGGCG | CAAAGCGACTAAGTTGGTTGAA |
| *BnaNLP2-1* | BnaA01g02150D | GTCCTTGACCAACAGAGTCATA | TCGGTGTCAAGGCAATTTTTAG |
| *BnaNLP2-2* | BnaA03g53210D | CGTTCATGAAGCTTCTCAACTC | CTTTGTCCTTTCAGGTAGCAAC |
| *BnaNLP2-3* | BnaC01g03280D | TTGGAAAGTGATGTCATGAAGC | GACAGTAAATGCCACACAGAAG |
| *BnaNLP2-4* | BnaC07g45500D | CGTTCATGAAGCTTCTCAAGTC | CACGTAGCAACCTTTCCATATG |
| *BnaNLP3-1* | BnaA06g40940D | CTTACTCGCTCAACCTCTTCTC | GTACTCCTCACTCCTGAAGAAG |
| *BnaNLP4-1* | BnaA06g14540D | CTGAGTTTCTCTTAGTTTGACCAG | GTCGAGAGCAAGATCCATAGAT |
| *BnaNLP4-2* | BnaA07g11340D | GCGAGTTTTAACCACAAAGGAA | GCTCCTGAAGAACCTAACATCT |
| *BnaNLP4-3* | BnaA08g21720D | ATGAGAGCAGCACTTTCTCTAG | CTGTCGAAGAACATCCAATGTG |
| *BnaNLP4-4* | BnaC05g16000D | GCTGATTTTGTGTTGGAGTTCT | CTCCTTATCCGTAACAGTCCTC |
| *BnaNLP4-5* | BnaC07g15260D | CCTAGTTCAAGCTCTAGCCAAT | CAATAGATCAGTTAACCTGCGC |
| *BnaNLP4-6* | BnaC08g19370D | ATGAGAGCAGCACTTTCTCTAG | GTGCTGTCGAAGAACATCAAAT |
| *BnaNLP5-1* | BnaA07g32630D | TGCAGAAAGGTCTAGTCTTGAG | TTCACCGCTTGAACTAATCTCT |
| *BnaNLP5-2* | BnaC06g37080D | CACAATAGACGATGCTTGCTAC | TAGCTAGAGACATCGGATGAGA |
| *BnaNLP6-1* | BnaA09g12180D | CATGATGTCACTCCGAACTTTG | GTGTACAAGTGAAGAGCATTGG |
| *BnaNLP6-2* | BnaCnng38990D | CTTCAAAGAATCAACTGGCCAA | AAGTGGTGAGAAGATATCGTCC |
| *BnaNLP7-1* | BnaA01g35090D | CAGCAACAACAACATAACGGTA | TACTTGATATCGAACGTACCGG |
| *BnaNLP7-2* | BnaA03g46410D | GAGTTTGTCCGACAACAATGAA | TTTAGTACTGTTTGGCCGTTTG |
| *BnaNLP7-3* | BnaC01g15850D | TTCGCTTCTTGGTTCTCTTTTG | GTCATCTTCACCAAAGTCAACC |
| *BnaNLP7-4* | BnaC07g38670D | CGCAGGTACGTTTGATATCAAG | TACAATTCTCCAGTACTCTCGC |
| *BnaNLP8-1* | BnaA03g20260D | GTTTGTCCCACTACACTGAAAC | CCGTATCTTCCTGACCTCTAAC |
| *BnaNLP8-2* | BnaA04g25110D | AAACGCTTTAGACTGCAAGAAG | CCAGCAGAACTACCTATACGAG |
| *BnaNLP8-3* | BnaA05g03380D | CGTACTTGAACTATTCTTGCCG | AACTTCGTTTTTAGTTGCTCCC |
| *BnaNLP8-4* | BnaC03g24230D | CCGCTACTGATATCTTGTTTGC | GTTAACTCATCACCATCGAACG |
| *BnaNLP8-5* | BnaC04g02980D | TGTAATATCTTCCCACGAGAGC | CGCAACTCCAGAACATTACAAG |
| *BnaNLP9-1* | BnaA07g18430D | GGAATGCTTTGTAAATGCATGC | GGTTTGTTTGATACGAGTGCTT |
| *BnaNLP9-2* | BnaC06g17440D | GATTCATCAAATGGTTCCGGAG | TACTCTCGCTGCTACTGTTATG |
| *BnaACT7* |  | TGGGTTTGCTGGTGACGAT | TGCCTAGGACGACCAACAATACT |
| *BnaUBC21* |  | CCTCTGCAGCCTCCTCAAGT | CATATCTCCCCTGTCTTGAAATGC |

All qRT-PCR primer sequences for *BnaNLP* genes were obtained from the qPrimerDB database (http://biodb.swu.edu.cn/qprimerdb).

Table S2 IDs and names of *NLP* genes in plants

| Gene ID | Gene name |
| --- | --- |
| GRMZM2G031398 | *ZmNLP8-1* |
| GRMZM2G031398 | *ZmNLP8-2* |
| GRMZM2G042278 | *ZmNLP7-1* |
| GRMZM2G048582 | *ZmNLP4-1* |
| GRMZM2G053298 | *ZmNLP4-2* |
| GRMZM2G105004 | *ZmNLP9-1* |
| GRMZM2G105004 | *ZmNLP9-2* |
| GRMZM2G109509 | *ZmNLP2-1* |
| GRMZM2G109509 | *ZmNLP2-2* |
| GRMZM2G176655 | *ZmNLP7-2* |
| GRMZM2G375675 | *ZmNLP6-1* |
| GRMZM2G466549 | *ZmNLP3* |
| GRMZM2G475305 | *ZmNLP6-2* |
| Sb03g000490 | *SbNLP6-1* |
| Sb01g048290 | *SbNLP2* |
| Sb02g031970 | *SbNLP5* |
| Sb04g002940 | *SbNLP6-2* |
| Sb06g021370 | *SbNLP9* |
| ERN06588 | *AmTrNLP7* |
| ERN20234 | *AmTrNLP8* |
| ERN04953 | *AmTrNLP6* |
| ERM96124 | *AmTrNLP5* |
| Gorai.009G187500 | *GorNLP4-1* |
| Gorai.005G013500 | *GorNLP8-1* |
| Gorai.004G131700 | *GorNLP6-1* |
| Gorai.011G138700 | *GorNLP8-2* |
| Gorai.002G115800 | *GorNLP4-2* |
| Gorai.005G126000 | *GorNLP8-3* |
| Gorai.003G074300 | *GorNLP6-2* |
| Gorai.006G206500 | *GorNLP6-3* |
| Gorai.008G041100 | *GorNLP1-1* |
| Bol023857 | *BolNLP1-1* |
| Bol013731 | *BolNLP2-1* |
| Bol026976 | *BolNLP4-1* |
| Bol025455 | *BolNLP4-2* |
| Bol027643 | *BolNLP5-1* |
| Os01g13540 | *OsNLP6-1* |
| Os11g16290 | *OsNLP8-1* |
| Os11g16290 | *OsNLP8-2* |
| Os03g03900 | *OsNLP2* |
| Os09g37710 | *OsNLP5-1* |
| Os09g37710 | *OsNLP5-2* |
| Os04g41850 | *OsNLP9-1* |
| Os02g04340 | *OsNLP6-2* |
| AT2G17150 | *ATNLP1* |
| AT4G35270 | *ATNLP2* |
| AT4G38340 | *ATNLP3* |
| AT1G20640 | *ATNLP4* |
| AT1G76350 | *ATNLP5* |
| AT1G64530 | *ATNLP6* |
| AT4G24020 | *ATNLP7* |
| AT2G43500 | *ATNLP8* |
| AT3G59580 | *ATNLP9* |
| Bra002025 | *BraNLP1-1* |
| Bra009659 | *BraNLP1-2* |
| Bra011600 | *BraNLP2-1* |
| Bra017705 | *BraNLP2-2* |
| Bra033561 | *BraNLP3-1* |
| Bra025834 | *BraNLP4-1* |
| Bra012241 | *BraNLP4-3* |
| Bra016458 | *BraNLP4-4* |
| Bra015758 | *BraNLP5-1* |
| Bra025834 | *BraNLP4-2* |
| Bra040914 | *BraNLP6-1* |
| Bra013754 | *BraNLP7-1* |
| Bra019251 | *BraNLP7-2* |
| Bra004767 | *BraNLP8-1* |
| Bra037711 | *BraNLP8-2* |
| Bra000307 | *BraNLP8-3* |
| Bra003382 | *BraNLP9-1* |
| Bra002025 | *BraNLP1-1* |
| Bol030007 | *BolNLP8-1* |
| BnaC07g06170D | *BnaNLP1-4* |
| BnaA01g02150D | *BnaNLP2-3* |
| BnaA03g53210D | *BnaNLP2-2* |
| BnaC01g03280D | *BnaNLP2-1* |
| BnaC07g45500D | *BnaNLP2-4* |
| BnaA06g40940D | *BnaNLP3-1* |
| BnaC03g47510D | *BnaNLP1-1* |
| BnaA07g03130D | *BnaNLP1-2* |
| BnaA06g25930D | *BnaNLP1-3* |
| BnaA07g11340D | *BnaNLP4-3* |
| BnaA08g21720D | *BnaNLP4-4* |
| BnaC05g16000D | *BnaNLP4-1* |
| BnaC07g15260D | *BnaNLP4-5* |
| BnaC08g19370D | *BnaNLP4-6* |
| BnaA01g35090D | *BnaNLP7-1* |
| BnaA03g46410D | *BnaNLP7-2* |
| BnaC01g15850D | *BnaNLP7-3* |
| BnaC07g38670D | *BnaNLP7-4* |
| BnaA03g20260D | *BnaNLP8-3* |
| BnaA07g18430D | *BnaNLP9-1* |
| BnaC06g17440D | *BnaNLP9-2* |
| BnaA06g14540D | *BnaNLP4-2* |
| BnaA07g32630D | *BnaNLP5-1* |
| BnaC06g37080D | *BnaNLP5-2* |
| BnaA07g21430D | *BnaNLP5-3* |
| BnaA09g12180D | *BnaNLP6-1* |
| BnaCnng38990D | *BnaNLP6-2* |
| BnaA04g25110D | *BnaNLP8-2* |
| BnaA05g03380D | *BnaNLP8-1* |
| BnaC03g24230D | *BnaNLP8-4* |
| BnaC04g02980D | *BnaNLP8-5* |
| BnaC04g48980D | *BnaNLP8-6* |
| BnaC08g29800D | *BnaNLP9-3* |
| Bol023805 | *BolNLP9-1* |
| Bol042145 | *BolNLP7-1* |
